# Case report of a young male, with recurrent pneumothorax, hemoptysis and intrapulmonary cavitary lesions

**DOI:** 10.1097/MD.0000000000035436

**Published:** 2023-10-06

**Authors:** Sun Junping, Sun Tianyu, Wang Rentao, Li Shengshu, Han Xiaobo, Zhang Xinxin, Zhang Mingyue

**Affiliations:** a Department of Respiratory and Critical Care Medicine, Chinese PLA General Hospital, Beijing, China.

**Keywords:** COL3A1, FLNB, spontaneous pneumothora, TSC2, vascular Ehlers-Danlos syndrome

## Abstract

**Rationale:**

Primary spontaneous pneumothorax (PSP) is a manifestation of Vascular Ehlers-Danlos syndrome (vEDS) caused by heterozygous mutations in the COL3A1 gene. vEDS is a rare inherited disorder with an prevalence of one in 150,000. It can causes PSP and severe fragility of connective tissues with arterial but it remains poorly defined on clinical grounds and diagnose. Through this report, we hoped to help clinicians further understand the characteristics of vEDS.

**Patient concerns:**

A 22-year-old man presented with recurrent pneumothorax, hemoptysis, and chest pain. Physical examination revealed remarkable hypermobility of the small joints and translucent skin with visible veins. Chest computed tomography (CT) showed pneumothorax and multiple pulmonary cavities.

**Intervention and outcome:**

Genomic deoxyribonucleic acid (DNA) was extracted from patients. Heterozygosity was observed in all 3 novel variants. The main variant is COL3A1, c.3256-43T > G(NM_000090.3), which represents a missense mutation in collagen type III alpha 1 that can lead to vEDS. The other 2 mutations were FLNB c.4814G > A(NM_001457.3) and TSC2 c.3145G > A (NM_000548.3). These variants were validated by Sanger sequencing of their parents. COL3A1was not detected in either of the parent strains. FLNB and TSC2 were detected in his mother.

**Diagnoses:**

Vascular Ehlers-Danlos syndrome.

**Lessons:**

Both COL3A1 and TSC2 gene mutations can cause PSP; however, to the best of our knowledge, there are no reports on these 2 gene mutations in 1 patient at the same time.

## 1. Introduction

Vascular Ehlers-Danlos syndrome (vEDS), also known as EDS type IV, is characterized by translucent skin, joint hypermobility, and a high risk of arterial rupture.^[1]^ The incidence of vEDS is approximately one in 150,000.^[2]^ COL3A1 gene heterozygous mutations can cause vEDS, which encodes the pro-α1 (III) chain of type III procollagen. Defects in the pro-alpha-1 III collagen chain are associated with vEDS. Ninety patients with vEDS present with external thoracic arterial dissection or rupture.^[1]^ vEDS is generally considered the most severe form of EDS.

Primary spontaneous pneumothorax (PSP) and pulmonary cysts are the manifestations of vEDS. Except for COL3A1, TSC2 mutations can also cause PSP, but there are no reports on these 2 gene mutations in 1 patient at the same time. In this report, we present a rare case, both COL3A1 and TSC2 gene mutations were appeared in this patien that manifested as recurrent pneumothorax, hemoptysis and intrapulmonary cavitary lesions.

## 2. Case presentation

**Figure 1.**
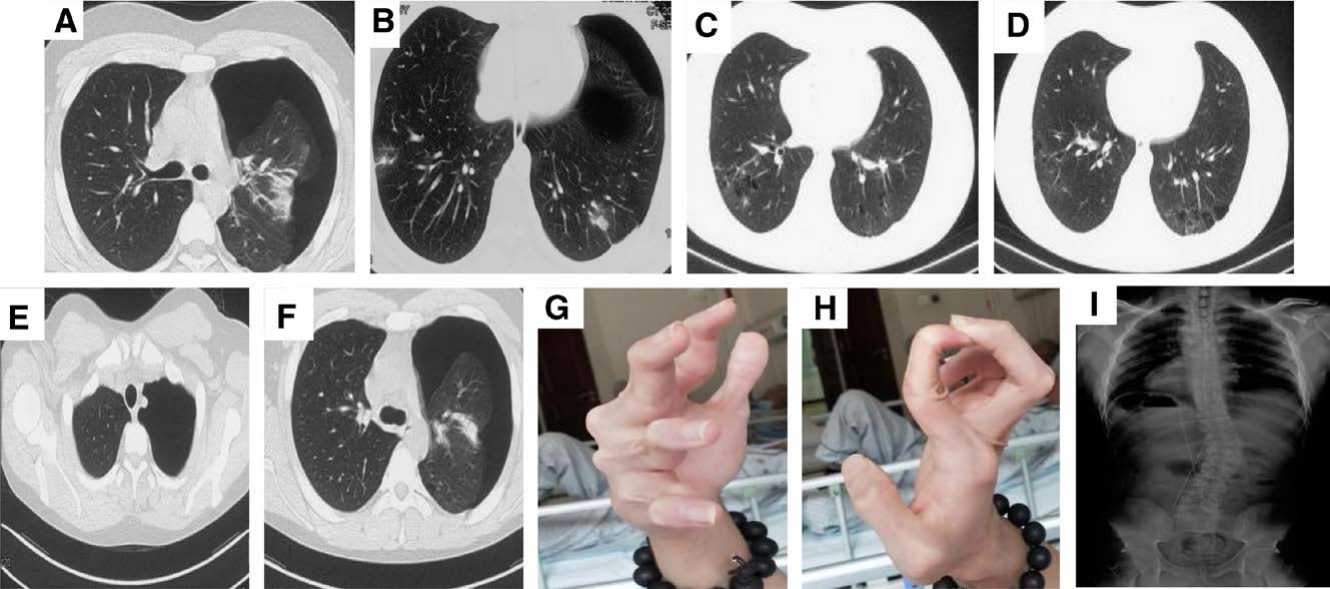
Chest CT showed left pneumothorax. Multiple pulmonary cavitary were found in the lower lobe. Nodules was observed in bilateral lower lobe (A–F). Mild hypermobility of the fingers was observed (G–H). X-ray of sacroiliac joint found his lower lumbar scoliosis was about 30° (I).

A 22-year-old man was admitted to our hospital with a recurrent pneumothorax, hemoptysis, and chest pain. Sixteen months earlier, the patient had discontinued chest pain and hemoptysis. Hemoptysis was stopped after 1 week. Three months later, these symptoms reappeared. Chest computed tomography (CT) showed left pneumothorax, and the lung was compressed by approximately 30%. Multiple pulmonary cavities are observed in the lower lobes. Nodules were observed bilaterally in the lower lobe (Fig. 1A and B). After 1 week of oxygen therapy (3 L/min, 24 h/d), the symptoms were relieved. Over the next 13 months, hemoptysis occurs intermittently and pneumothorax recurred 3 times due to coughing, sneezing and exertion in defecation, all on the left.He had a history of hypertension and was born with scoliosis and varus. Betaloc was used to control blood pressure and was maintained within the normal range.

Physical examination revealed a temperature of 36.3°C, heart rate of 80 beats/min, blood pressure of 120/80 mm Hg, and respiratory rate of 19 breaths/min. The body mass index was 27.64 kg/m^2^. His skin was thin and translucent, with visible veins, especially in the chest and limbs. Mild finger hypermobility was observed (Fig. 1G and H). Chest auscultation revealed decreased lung sounds in the left hemithorax.

Laboratory tests revealed normal routine blood, liver, and kidney function. The erythrocyte sedimentation rate and C-reactive protein level were within the normal limits. Tests for connective tissue disease with auto antibodies, including antinuclear, anti CCP and anti-neutrophil cytoplasmic were negative. G test, GM test and Cryptococcus capsular antigen were all negative. The test for human immunodeficiency virus was negative. Immuno-fix electrophoresi and light chain detection were also negative.

Subsequent chest CT (Fig. 1C–F) revealed left-sided pneumothorax and several small cavitary lesions in the bilateral lobes and nodules in the right lower lobe. An intercostal chest drain was inserted with complete resolution of pneumothorax. The patient underwent bronchoscopy, which revealed a small amount of bleeding at the opening of the upper lobe of the right lung; no obvious abnormalities were found in the rest. Radiography of the sacroiliac joint revealed lower lumbar scoliosis of approximately 30° (Fig. 1I). The structure and function of the heart were normal. No abnormalities were found in the liver, gallbladder, pancreas, spleen, adrenal gland, bilateral renal arteries or abdominal aorta. Abdomen enhanced CT showed no obvious abnormality. Brain MRI suggested that developmental venous malformation near the posterior horn of the left ventricle, but no obvious abnormality was found in Computed Tomography Angiography of neck and brain.

For clear diagnosis, genomic deoxyribonucleic acid was extracted from the blood samples of the patient and his parents. Whole exome sequencing analysis was performed on the deoxyribonucleic acid samples using chip capture high-throughput sequencing at the Shenzhen Huada Clinical Laboratory Center, Shen Zhen, China. Chip-capture high-throughput sequencing was performed to sequence the exons and adjacent exon sequences of approximately 20,000 genes in the human genome. A variant analysis was performed on 3583 genes in the Online Mendelian Inheritance in Man database, which showed a clear correlation with a single genetic disease. The process of analysis involved first filtering the sequences by 1% of the population frequency (variants present in greater than 1% of the population frequency were evaluated as sites with a low probability of etiology). On the basis of the patients’ clinical complaints, we assessed whether the mutation sites were likely to fit the symptoms in the filtered mutation dataset. The pathogenicity of the variants was interpreted and classified according to the American College of Medical Genetics (ACMG) guidelines published in 2015 and the sequence variation guidelines of the American Society of Molecular Pathology (AMP).

**Figure 2.**
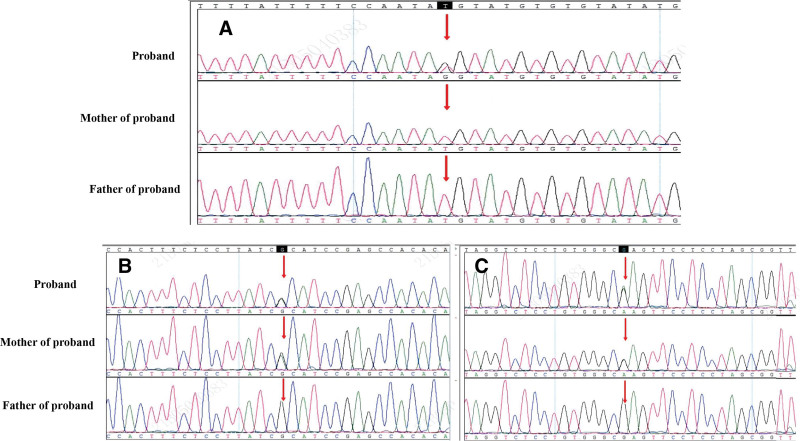
Heterozygosity was observed 3 novel variant. The main variant is COL3A1, c.3256-43T > G (NM_000090.3), Sanger sequencing showed COL3A1 c.3256-43T > G (NM_000090.3) mutation (A) The other 2 mutations are FLNB c.4814G > A (NM_001457.3) represented bone dysplasia and TSC2 c.3145G > A (NM_000548.3). Sanger sequencing showed FLNBc.4814G > A (NM_001457.3) mutation (B). Sanger sequencing showed TSC2c.3145G > A (NM_000548.3) mutation (C). COL3A1was not detected in either of his parents. FLNB and TSC2 were detected in his mother.

Heterozygosity was observed in 3 novel variants: COL3A1, c.3256-43T > G(NM_000090.3), (Fig. 2A) which represented a missense mutation in the collagen type III alpha 1 protein; the other 2 mutations were FLNB c.4814G > A (NM_001457.3) (Fig. 2B) represented bone dysplasia; and TSC2 c.3145G > A (NM_000548.3) (Fig. 2C) represented tuberous sclerosis complex. The mutations were named according to the recommendations of the Human Genome Variation Society (HGVS) recommendations. These variants were validated by Sanger sequencing of the parents. COL3A1was not detected in either parent, indicating that this variant was a de novo mutation. FLNB and TSC2 were detected in his mother. His mother was healthy and no abnormalities have been found to date.

## 3. Discussion

PSP is defined as the spontaneous occurrence of pneumothorax in a healthy individual.^[3]^ A positive family history was found in 11.5% of patients.^[4]^Genetic contributions to both sporadic and familial pneumothorax. Mutations and deletions in FBN1, COL3A1, CBS, SERPINA1, TSC1, TSC2, and FLCN have been attributed to PSP.^[5]^ At present, there is no literature reporting that patients with PSP have both COL3A1 and TSC2 mutations simultaneously. This is the first report.

COL3A1 provides instructions for the production of type III collagen. Type III collagen is found in skin, lungs, intestinal walls, and blood vessels. Abnormal type III collagen synthesis is associated with hyperextensibility of the skin, joint hypermobility, and increased tissue fragility.^[6,7]^ Thin and translucent skin with visible veins and mild hypermobility of the fingers was observed in our patient. COL3A1 mutations can result in reduced production of mature type III collagen and reduced mechanical strength of arteries and other hollow organs,^[7]^ leading to complications such as arterial complications, aneurysm formation, or spontaneous rupture of hollow organs, most frequently in the uterus or intestines. However, these complications were not observed in this study.

vEDS mostly affects the lungs. One study found that approximately 17.7% of the patients with vEDS had lung involvement. The most common respiratory complication is pneumothorax, and the incidence rate of respiratory tract infections is higher than that in ordinary people. Hemoptysis and spontaneous hemothorax are the common manifestations of vEDS. Patients with vEDS often present with cavitary or bullous lung disease, emphysema, pulmonary cysts, or bronchiectasis. Spontaneous pneumothorax and hemoptysis may be initial manifestations of vEDS.

The patient also harbored a TSC2 c.3145G > A mutation. More than 10,000 individuals with TSC and their families in whom pathogenic variants have been identified, 26% of probands have a pathogenic variant in TSC1 and 74% have a pathogenic variant in TSC2.^[8]^ To date, this mutation has been reported as pathogenic. Multitudinous clinical findings are possible in TSC affecting the brain, skin, abdominal viscera, heart, eyes, mouth and lung.^[9]^ Both COL3A1 and TSC2 mutations can cause PSP. TSC2 mutations can cause LAM (lymphangioleiomyomatosis). Among patients with LAM, patients with TSC (TSC-LAM) make up 15%.^[10]^ However, LAM is typically diagnosed in young adults and affects almost exclusively females, a very small number of men have LAM. Both COL3A1 and TSC2 gene mutations appeared in this patient. The limitation of this case is that there is no way to determine exactly which of the 2 gene mutations cause patient’s current situation. But his mother had a TSC2 mutation ans she was a normal person and no abnormalities have been found in her lungs so far. Therefore, we believe that the occurrence of pneumothorax, changes in lung CT, and various clinical symptoms are caused by the COL3A1 gene mutation.

Here, we report a case of a patient with a novel point mutation in Filamin B (FLNB). Using dideoxy sequencing, FLNB c.4814G > A encoding p. (Arg1605His). However, there are no reports on the pathogenicity of this mutation. It has high histological specificity for the skeletal system. FLNB is a large dimeric actin-binding protein that cross-links actin cytoskeleton filaments into a dynamic structure.^[11]^ Pathogenic mutations in FLNB have been found to cause skeletal deformities and play an important role in skeletal development.^[12]^ COL3A1 mutations can cause small joint hyperflexion, clubfoot, chronic joint dislocation or subluxation. The patient had foot varus and scoliosis at the time of birth. Based on the disease monism, we consider that it is related to the COL3A1 mutation; however we cannot exclude the influence of FLNB mutations.

## 4. Conclusion

In young patients with recurrent pneumothorax and hemoptysis of unclear cause, the diagnosis of vEDS should be considered, and genes should also be screened for differential diagnosis.

## Author contributions

**Conceptualization:** Sun Junping, Sun Tianyu, Wang Rentao.

**Data curation:** Sun Junping.

**Investigation:** Sun Junping, Sun Tianyu, Wang Rentao, Li Shengshu.

**Resources:** Sun Junping, Sun Tianyu, Wang Rentao, Li Shengshu, Han Xiaobo, Zhang Xinxin.

**Visualization:** Sun Junping.

**Writing – original draft:** Sun Junping, Sun Tianyu, Wang Rentao, Zhang Mingyue.

**Writing – review & editing:** Sun Junping, Wang Rentao.
